# Supplementary material for: Hazard Identification Related to the Presence of Vibrio spp., Biogenic Amines, and Indole-Producing Bacteria in a Non-Filter Feeding Marine Gastropod (Tritia mutabilis) Commercialized on the Italian Market
Source: Foods. 2021 Oct 25;10(11):2574. doi: 10.3390/foods10112574 (PMC8617628; doi:10.3390/foods10112574)
Supplement: Supplementary file 1 [file foods-10-02574-s001.zip › foods-1400342-supplementary.pdf]

Table S1. Target genes and oligonucleotide primers utilized in the study.

Vibrio species	Target Gene Specie/Virulence [a]	Primersb	Sequence	Ampl (bp)	References[c]
V. cholera	*tox*R-C (S)	toxR-F<br>toxR-R	CCTTCGATCCCCTAAGCAATAC<br>AGGGTTAGCAACGATGCGTAAG	779	Rivera et al., 2001[c]
	*ctx*A (V)	ctxA-F<br>ctxA-R	CGGGCAGATTCTAGACCTCCTG<br>CGATGATCTTGGAGCATTCCCAC	564	Fields et al., 1992[d]
	*ctx*B (V)	ctxB-F<br>ctxB-R	GGTTGCTTCTCATCATCGAACCAC<br>GATACACATAATAGAATTAAGGATG	460	Olsvik et al. (1993)[e]
	stn/sto (V)	stn/sto67-F<br>stn/sto194-R	GCTGGATTGCAACATATTTCGC<br>TCGCATTTAGCCAAACAGTAGAAA	172	Rivera et al., 2001[c]
V. parahaemolyticus	*tox*R-P (S)	toxR-F<br>toxR-R	GTCTTCTGACGCAATCGTTG<br>ATACGAGTGGTTGCTGTCATG	368	Kim et al., 1999[f]
	tdh (V)	tdh-F<br>tdh-R	GTAAAGGTCTCTGACTTTTGGAC<br>TGGAATAGAACCTTCATCTTCACC	269	Bej et al., 1999[g]
	trh (V)	trh-F<br>trh-R	TTGGCTTCGATATTTTCAGTATCT<br>CATAACAAACATATGCCCATTTCCG	500	Bej et al., 1999 [g]
V. vulnificus	*vvh*A (S)	Vvh785-F<br>Vvh1303-R	CCGCGGTACAGGTTGGCGCA<br>CGCCACCCACTTTCGGGCC	519	Han & Ge, 2010[h]

[a] Specie markers (S), virulence markers (V);

[b] Oligonucleotide primers and PCR conditions: a small amount of the purified strain was picked from the plate and transferred in 100 µL of sterile H2O. The DNA was extracted by the boiling method at 100°C for 15 min, stored 2-3 min at -20°C and then centrifuged at 5000 × g at 4°C for 15 min, recovering the supernatant. Bacterial lysates were stored at -20°C until use. For the PCR reactions 1 µL of each lysate was suspended with 2.5 µL 10X PCR Rxn Buffer (Invitrogen, Carlsbad, CA, USA), 1 µL MgSO4 (Invitrogen), 0.5 µL dNTPs 100 Mm (Invitrogen), 1 µL of each primer, 0.2 µL of Taq-DNA Polymerase (Invitrogen), and sterile H2O to the mark of 25 µL. PCR reactions were electrophoresed at 100 V for 30 min on 1.5% agarose gel utilizing tris-acetate-ethylenediaminetetraacetic acid (TAE) buffer (Sigma Aldrich, St. Louis, MO, USA); Gel Red Nucleic Acid Stain (Biotium, Hayward, CA, USA) was utilized as fluorescent nucleic acid stain, and bromophenol blue with 30% glycerol in PCR water and TrackIt™ 100 bp DNA Ladder (Invitrogen) as loading buffer. The PCR products were evidenced by the transilluminator Bio-Rad Gel Doc™ XR, Trans-UV at 302 nm;

[c] Rivera, I.N.; Chun, J.; Huq, A.; Sack, R.B.; Colwell, R.R. Genotypes Associated with Virulence in Environmental Isolates of Vibrio cholerae. A*ppl. Environ. Microb.* **2001,** *67*, 2421-2429, https://doi: 10.1128/AEM.67.6.2421-2429.2001; [d] Fields, P.I.; Popovic, T.; Wachsmuth, K.; Olsvik, O. Use of polymerase chain reaction for detection of toxigenic Vibrio cholerae O1 strains from the Latin American cholera epidemic. *J. Clin. Microbiol.* **1992,** *30*, 2118-2121, https://doi: 10.1128/jcm.30.8.2118-2121.1992; [e] Olsvik, O.; Wahlberg, J.; Petterson, B.; Uhlén, M.; Popovic, T.; Wachsmuth, I.K.; Fields, P.I. Use of automated sequencing of polymerase chain reaction-generated amplicons to identify three types of cholera toxin subunit B in Vibrio cholerae O1 strains. *J. Clin. Microbiol.* **1993**, *31*, 22–25, https://doi: 10.1128/jcm.31.1.22-25.1993; [f] Kim, Y.B.; Okuda, J.; Matsumoto, C.; Takahashi, N.; Hashimoto, S.; Nishibuchi, M. Identification of Vibrio parahaemolyticus strains at the species level by PCR targeted to the toxR gene. *J. Clin. Microbiol.* **1999,** *37*, 1173-1177, https://doi: 10.1128/JCM.37.4.1173-1177.1999; [g] Bej, A.K.; Patterson, D.P.; Brasher, C.W.; Vickery, M.C.; Jones, D.D.; Kaysner, C.A. Detection of total and hemolysin-producing Vibrio parahaemolyticus in shellfish using multiplex PCR amplification of tl, tdh and trh. *J. Microbiol. Meth.* **1999,** *36*, 215-225, https://doi: 10.1016/s0167-7012(99)00037-8; [h] Han, F.; Ge, B. Multiplex PCR assays for simultaneous detection and characterization of Vibrio vulnificus strains. *Lett. Appl. Microbiol.* **2010,** *5*, 234-240. https://doi: 10.1111/j.1472-765X.2010.02887.x.
